# Effectiveness of contact-based education for reducing mental illness-related stigma in pharmacy students

**DOI:** 10.1186/1472-6920-12-120

**Published:** 2012-12-05

**Authors:** Scott B Patten, Alfred Remillard, Leslie Phillips, Geeta Modgill, Andrew CH Szeto, Aliya Kassam, David M Gardner

**Affiliations:** 1 Community Health Sciences, University of Calgary, 3rd Floor TRW Bldg., 3280 Hospital Drive NW, Calgary, AB, Canada; 2 College of Pharmacy and Nutrition, University of Saskatchewan, Saskatoon, SK, Canada; 3 School of Pharmacy, Memorial University of Newfoundland, St. John’s NL, Canada; 4 Mental Health Commission of Canada, Calgary, Canada; 5 Mental Health Commission of Canada and Department of Psychology, University of Calgary, Calgary, AB, Canada; 6 Post Graduate Medical Education and Medical Education Research Group, University of Calgary, Calgary, AB, Canada; 7 Department of Psychiatry and College of Pharmacy, Dalhousie University, Halifax, NS, Canada

## Abstract

**Background:**

A strategy for reducing mental illness-related stigma in health-profession students is to include contact-based sessions in their educational curricula. In such sessions students are able to interact socially with a person that has a mental illness. We sought to evaluate the effectiveness of this strategy in a multi-centre study of pharmacy students.

**Methods:**

The study was a randomized controlled trial conducted at three sites. Because it was necessary that all students receive the contact-based sessions, the students were randomized either to an early or late intervention, with the late intervention group not having participated in the contact-based education at the time when the primary outcome was assessed. The primary outcome, stigma, was assessed using an attitudes scale called the Opening Minds Survey for Health Care Providers (OMS-HC).

**Results:**

We initially confirmed that outcomes were homogeneous across study centres, centre by group interaction, p = 0.76. The results were pooled across the three study centres. A significant reduction in stigma was observed in association with the contact-based sessions (mean change 4.3 versus 1.5, t=2.1, p=0.04). The effect size (Cohen’s d) was 0.45. A similar reduction was seen in the control group when they later received the intervention.

**Conclusions:**

Contact-based education is an effective method of reducing stigma during pharmacy education. These results add to a growing literature confirming the effectiveness of contact-based strategies for stigma reduction in health profession trainees.

## Background

Although there is no generally accepted ‘unitary theory’ of stigma [1], it can be defined as ‘an attribute that is deeply discrediting and that reduces the bearer from a whole and usual person to a tainted, discounted one’ [2]. A sparsely studied yet significant area of concern is stigma and discrimination against people with mental illness by health care providers. Attitudes held by health care providers can have a negative impact on patient quality of life [3].

Stigmatizing attitudes or behaviours by pharmacists have the potential to undermine the patient-pharmacist relationship and are likely to compound a mentally ill person’s feelings of rejection and isolation. A primary goal of the pharmacist is to identify and resolve patient-specific medication-related issues, ensuring treatment choices are accepted, needed, safe and effective [4]. To achieve this goal, the pharmacist must gather information from the patient about health and medication concerns, other mental or physical health problems, use of other medications or substances, and so on. Pharmacist attitudes and communication styles (verbal and non-verbal) are pivotal in establishing the strong therapeutic rapport required to achieve these goals [4]. Involvement of pharmacists in mental health care can improve outcomes, prescribing practices, patient satisfaction, and resource use [5].

The stigmatization of people with mental illness by pharmacists has the same negative effects propagated by other groups within the health care team [6]. Stigma by pharmacists may lead to counterproductive communications and failure to meet the health care needs of the individual [7]. For example, a stigmatizing pharmacist may be less likely to inform a young woman who self-selects St John’s wort for depression that the herbal medication can reduce the effectiveness of her oral contraceptives [8]. The same pharmacist may appear to be less approachable by a craft worker who is frustrated by fine hand tremor caused by lithium and is considering stopping it.

A recent study by Black et al. [9] assessed patient preferences, satisfaction and perceived stigma by community pharmacists. Of concern is that 26% indicated they did not feel comfortable speaking to a pharmacist about their mental health medication. This suggests that stigma may play a significant role in preventing patients from achieving the maximum benefit from professional pharmacy services.

Phokeo et al. [7] examined community pharmacists’ attitudes toward and professional interactions with users of psychiatric medication. A greater proportion of pharmacists expressed discomfort discussing symptoms and medications with patients who had mental illness (36%) compared to patients with cardiovascular disease (6%). Scheerder et al. [10] reported discrepancies in pharmacists’ practice involving depressed patients when compared to other conditions. For example, 70% of pharmacists reported maintaining a trusting relationship with most or all patients with other conditions but only 32% reported this for depressed patients. In a survey of Belgian pharmacists, Liekens et al. [11] found that even though pharmacists held generally positive attitudes toward providing care for people with depression, they also reported delivering less care. Based on data collected in a mail survey, Rickles et al. [12] reported that pharmacists had a lower willingness to provide services for mental illness versus asthma.

Negative attitudes among pharmacy students were reported in a series of international surveys, although specific attitudes were found to vary considerably by country [13].

A study by Bell et al., [14] compared the attitudes of third year pharmacy students with those of pharmacy graduates. The third year students had not yet received any mental health lectures or tutorials as part of their curriculum whereas the graduates had completed lectures and tutorials as well as six months of supervised clinical practice. Stigmatizing attitudes towards people with mental illness were documented in both groups and there was no statistical difference noted between groups. These results suggest that traditional forms of pharmacy education do not reduce stigma. Consistent with this, Jermain and Crismon [15] reported that psychiatric rotations did not improve social distance ratings among pharmacy students (a preference for greater social distance is generally viewed as a component of stigmatization). Another study that included an assessment of social distance reported improved ratings in 60 pharmacy students that participated in a mental health first aid course, compared to 212 students that either did not apply for the course or were not randomly selected for it.

Involvement of consumer-educators in pharmacy education has been viewed as a possible means to diminish stigma. A non-randomized study by Bell et al. [16] compared the impact of a tutorial session involving a pharmacist instructor, small group work, case studies and role playing when the tutorial was delivered with and without participation of a consumer educator. After the intervention, students in the consumer-educator group showed more consistent improvement on items indicative of social distance, diminished negative attitudes and increased positive attitudes towards mental illness.

At least three Canadian pharmacy faculties have incorporated measures to address stigma into their curricula. These schools have made use of contact-based learning interventions for several years. Contact-based education uses social contact as a way to improve relations among groups that are experiencing stigma and discrimination [17,18]. Stigma is reduced by providing an opportunity for interpersonal contact between people who have a mental illness and audiences who may be stigmatizing towards them. A key ingredient of contact-based education is the delivery of testimonies by service users [19]. The success of contact-based education is generally supported in the literature, however, the type of contact has to be appropriate [17,18]. That is, the contact must be with people who live with a mental illness but are recovered or in recovery.

The purpose of this study was to evaluate the impact of contact-based education on pharmacy students’ attitudes towards people with mental illness. It was hypothesized that contact-based sessions would diminish stigma towards individuals with mental illness.

## Methods

### Study design

This was a randomized trial that involved 3^rd^ and 4^th^ year pharmacy students enrolled at three Canadian undergraduate pharmacy programs (Dalhousie University, Memorial University of Newfoundland, and the University of Saskatchewan). For several years, each of these institutions has offered a contact-based session (see below for a description) ranging from 60 to 90 minutes as part of their mental health curriculum. In order to differentiate whether this specific aspect of the curriculum was effective at reducing stigma, students were randomized (1:1) to either receive the intervention, or not, early in their mental health unit. Those not receiving the intervention early participated at a later time, near the end of the unit, approximately 2–3 weeks later. Stigma was assessed at three time points: (1) prior to any students receiving the intervention, (2) when the early group had attended the session and the late group had not, and (3) after both groups had received the intervention. The primary analysis was assessed at the second time point and evaluated change from the baseline assessment to that time point.

**Figure 1:**
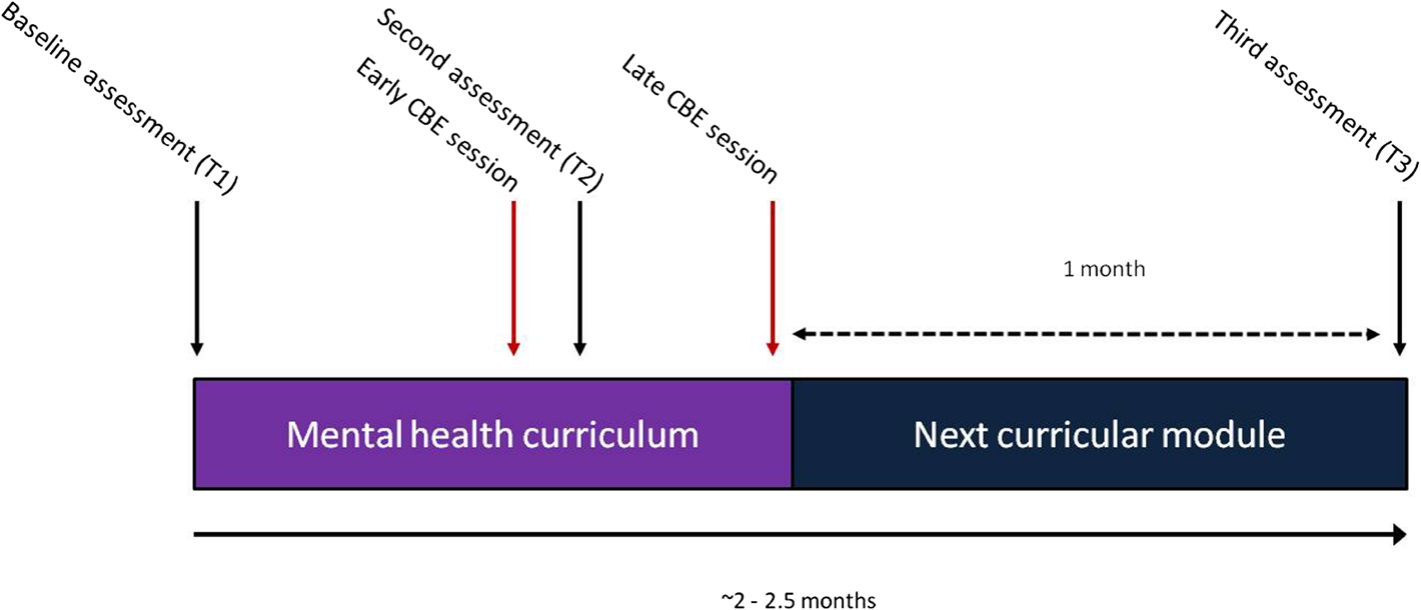
Schematic of study procedures*. *CBE = Contact Based Education.

For operational reasons, the T3 assessment occurred approximately one month after the T2 assessment, such that there was a longer duration of time between the intervention and post-intervention assessment in the late group. See Figure 1 for a schematic representation of the study design. Data for the second assessment was collected 2–3 days after the session at Dalhousie, where the students deposited their questionnaire into a box (preserving their anonymity). At the other schools an on-line system was used to collect the data, generally within a similar time frame. Participation was highest at Dalhousie University. For this reason, we conducted a sensitivity analysis with exclusion of the lower-response rate sites.

All students participated in a mental health lecture series and case discussions during their mental health rotations. At Dalhousie the curriculum is problem based, and for 5 weeks the focus is mental health and psychotropic medications. At Memorial, the rotation involved 32 hours of instructional time, 18 hours of lecture and 12 hours of tutorials over approximately 4.5 weeks. The Saskatchewan rotation consisted of 20 hours of lectures and 2 ninety minute tutorials. No compensation was provided to participants in the study. None of the rotations involved clinical placements.

There were 84 students eligible to participate from Dalhousie University, 86 from the University of Saskatchewan, and 41 students from Memorial University. All students were asked to complete a baseline questionnaire before their mental health rotation began. Those randomly allocated to the “early group” completed the second questionnaire following their contact-based education session. At the same time the “late group” also completed this questionnaire but without having yet experienced the contact-based education. The study design was possible because of flexibility in the curricula at these schools. While the early group was doing their contact-based education sessions, the late group was involved in other aspects of their curriculum, and vice versa. The educational content did not differ between the groups, only the order in which different educational activities were delivered. Ethics approval for the study was received from the Conjoint Health Research Ethics Board at the University of Calgary, and from Ethics Boards at each of the three sites. Data collection occurred between September 2010 and May 2011.

### Intervention: contact-based education

The contact-based education involved learning about mental illness from people with first-hand experience. Each of the contact-based sessions employed people with lived experience, but who were in a state of recovery. None of the sessions had a lecture format and all were designed to foster interaction between the students and the person with lived experience. In keeping with this goal, all of the sessions were interactive. At Dalhousie University, each of two 1 hour contact based-educational sessions involved two young adults with a diagnosis of bipolar disorder or schizophrenia. The early and late sessions involved different people, due to limits on availability, addressing approximately 42 students per session. Speakers told their personal stories (provided testimonies) and answered student questions. Each session was facilitated by the course professor and a staff member of a local mental health support organization. Questions of “have you ever experienced stigma in a pharmacy?” and “what do you want from your pharmacist?” were addressed among other more personal questions about living with a mental illness.

At the University of Saskatchewan, the early and late sessions were 90-minutes in duration and involved the same three speakers to ensure consistency. Two speakers indicated that their diagnoses were schizophrenia and schizoaffective disorder. They were also in a state of recovery at the time of the presentation. They spoke about their experience growing up with a mental illness and their experiences with its management. The other speaker was a mother of a young man with schizophrenia who spoke about what she and the family members experienced in relation to her son’s mental illness. The session was complemented by the input of a mental health professional that provided an overview on schizophrenia and its management.

Similarly, at Memorial, three members of the Newfoundland Chapter of the Schizophrenia Society (patient, family member, and a health care provider) delivered a two hour session on their experiences with this illness. The sessions were facilitated by a course instructor and included discussions about the types of interactions and resultant impressions the guest speakers had formed regarding their experiences with pharmacists.

### Survey instrument

A 20-item questionnaire, the Opening Minds Survey for Health Care Providers (OMS-HC), was the instrument used to assess attitudes of pharmacy students toward people with mental illness (Additional file 1). The OMS-HC requires participants to respond to statements using a 5-point Likert scale from “strongly agree” to “strongly disagree”. Each item is scored 1–5, such that OMS-HC scores range from 20 (minimal stigma) to 100 (maximum stigma). The OMS-HC was developed and validated in an earlier study [20]. Validation procedures included the development, testing and refinement of an item-pool, obtaining feedback from experts and focus groups and psychometric evaluation. Psychometric analyses indicated that the OMS-HC scale can be scored using a single total score, but that it encompasses five stigma domains: social distance, disclosure, self-stigma, prejudice and devaluation, and the social responsibility and role of health care providers. The overall scores provide a global index of stigmatization. Its internal consistency in the development phase was found to be good (Cronbach’s alpha = 0.82) with satisfactory test-retest reliability, intraclass correlation = 0.66 (95% CI 0.54 to 0.75). The OMS-HC was only weakly correlated with social desirability, indicating that the social desirability bias was not likely to be a major determinant of OMS-HC scores.

Some of the questions on the OMS-HC survey instrument were adapted from existing scales and some were original questions. Four questions were used from the Mental Illness: Clinicians’ Attitudes (MICA) scale [21]. The MICA scale was developed for use with medical students in England and has been shown to have satisfactory validity and reliability. It is responsive to change before and after stigma intervention. Several additional items were used in the data collection as well, including demographic information (age and gender) and an item asking whether participants had a close friend or family member with a mental illness. As social contact may diminish stigma, social contact occurring within family or peer groups was a possible confounder. Demographic variables needed to be measured as they also had the potential to confound or modify the results.

### Data analysis

The primary outcome variable was change in OMS-HC score between baseline and the second survey (T1 to T2), at which time only the early group had experienced the contact-based education. Strictly speaking the primary analysis was not based on intention to treat as it required that there be two measures in order to be included. The change scores were found to be approximately normally distributed and had similar variance in the two groups.

All analyses used Stata [22]. Descriptive statistics were calculated for demographic variables and baseline OMS-HC scores. Prior to comparing the two groups, we used an analysis of centre by group interactions to confirm that the effects of the intervention were homogeneous across centres. Initially, change in OMS-HC scores were compared using an independent samples *t*-test. The possibility of differential intervention effects by age and gender were explored using interaction terms. Effect sizes using Cohen’s d approach were calculated for OMS-HC change scores.

In a secondary analysis we used a mixed-model to adjust confidence limits for possible correlations within study sites and to incorporate the T3 data [22]. Two observations (change from baseline to the second survey and change from the second to the third survey) were nested within each subject, and the subjects were nested within their study centre. The success of the intervention was assessed using the Wald test of interaction between group and interval (T1 to T2 and T2 to T3).

## Results

### Sample characteristics

Of the 211 (Dalhousie, *n =* 84; Saskatchewan, *n =* 86; Memorial, *n =* 41) students eligible to participate in the study, 131 completed a baseline survey (62.1% response rate). Of these, 87 students completed the second survey and 74 completed all three surveys yielding follow-up rates of 66.4% (T2) and 56.5% (T3).

### Baseline measures

The largest proportion of respondents completing the baseline survey were from Dalhousie University (49.6%) followed by the University of Saskatchewan (26.7%) and Memorial University (23.7%). Participant characteristics are summarized in Table 1. The two right-hand columns of this table present frequencies among subjects with complete data collection by the second (T2) and final (T3) time points. The higher rate of successful follow-up at Dalhousie was the only significant difference between completers and non-completers, see Table 1.

**Table 1:** Description of respondents with and without complete follow-up

	**Baseline (T1) All respondents (*n*=131)**	**Baseline Incomplete Follow-up (n=57)**	**Baseline Complete Follow-up (n=74)**	p-value*	**T1 to T2 (*n = *87)**	**T1 to T3 *(n = *74)**
Group						
Early	58 (44.3 %)	28 (49.1 %)	30 (45.5 %)	0.38	36 (41.4 %)	30 (45.5 %)
Late	73 (55.7 %)	29 (50.9 %)	44 (59.5 %)		51 (58.6 %)	44 (59.5 %)
School						
Memorial	31 (23.7 %)	21 (36.8 %)	10 (13.5 %)	P<0.001	13 (14.9 %)	10 (13.5 %)
Saskatchewan	35 (26.7 %)	21 (36.8 %)	14 (18.9 %)		18 (20.7 %)	14 (18.9 %)
Dalhousie	65 (49.6 %)	15 (26.3 %)	50 (67.6 %)		56 (64.4 %)	50 (67.6 %)
Sex						
Male	35 (26.7 %)			0.55	21 (24.1 %)	18 (24.3%)
Female	96 (73.3 %)				66 (75.9 %)	56 (75.7%)
Age, Mean (sd)	23.8 (2.6)	23.7 (1.9)	24.0 (3.1)	0.53	23.9 (2.9)	24.0 (3.1)
Close friend/ family with MI*						
No	11 (8.9 %)	4 (7.3 %)	7 (10.3 %)	0.93	9 (11.2 %)	7 (10.3 %)
Yes	105 (85.4 %)	48 (87.3 %)	57 (83.8 %)		67 (83.8 %)	57 (83.8 %)
Don’t know	7 (5.7 %)	3 (5.5 %)	4 (5.9 %)		4 (5.0 %)	4 (5.9 %)

* Comparison of respondents with and without complete follow-up. Fisher’s exact test was used for categorical variables and a t-test was used in the comparison of mean age.

** Not all participants responded to this item. MI: mental illness.

The mean age of the respondents was 23.8 years (standard deviation 2.6) and 73% of respondents were female. As seen in Table 2, there was no major difference between early and late intervention groups, per randomization. None of the baseline differences seen in Table 2, were statistically significant (p-values not shown, all > 0.05). Most respondents (85.4%) indicated knowing a family member or close friend with a mental illness.

**Table 2:** Description of respondents, by group

	**All respondents (*n *= 131)**		**T1 to T2 (*n *= 87)**		**T1 to T3 (*n *= 74)**	
	**early (*n *= 58)**	**late (*n *= 73)**	**early (*n *= 36)**	**late (*n *= 51)**	**early (*n *= 30)**	**late (*n *= 44)**
School						
Memorial	13 (22.4 %)	18 (24.7 %)	3 (8.3 %)	10 (19.6 %)	2 (6.7 %)	8 (18.1 %)
Saskatchewan	16 (27.6 %)	19 (26.0 %)	8 (22.2 %)	10 (19.6 %)	6 (20.0 %)	8 (18.1 %)
Dalhousie	29 (50.0 %)	36 (49.3 %)	25 (69.4 %)	31 (60.8 %)	22 (73.3 %)	28 (63.6 %)
Sex						
Male	16 (27.6 %)	19 (26.0 %)	8 (22.2 %)	13 (25.5 %)	7 (23.3 %)	11 (25.0 %)
Female	42 (72.4 %)	54 (74.0 %)	28 (77.8 %)	38 (75.5 %)	23 (76.7 %)	33 (75.0 %)
Age, mean (sd)	23.6 (1.7)	24.1 (3.3)	23.7 (2.0)	24.1 (3.4)	23.7 (2.0)	24.2 (3.6)
Close friend/family with MI**						
No	6 (10.9 %)	5 (7.4 %)	6 (17.7 %)	3 (6.5 %)	5 (17.2 %)	2 (5.1 %)
Yes	43 (78.2 %)	62 (91.2 %)	24 (70.6 %)	43 (93.5 %)	20 (69.0 %)	37 (94.9 %)
Don’t know	6 (10.9 %)	1 (1.5 %)	4 (11.8 %)	0 (0 %)	4 (13.8 %)	0 (0 %)

* there were no significant differences between early and late groups. Fisher’s exact test was used for categorical variables and a *t*-test was used in the comparison of mean age.

** totals for “no”, “yes” and “don’t know” responses do not sum to the column totals due to missing data on this item. MI: mental illness.

### Outcomes

The internal consistency of the OMS-HC in this sample, measured using Cronbach’s alpha, was 0.84 at baseline, 0.85 at T2 and 0.86, at T3. We initially assessed the homogeneity of the intervention effect across study sites by assessing group by centre interaction. As there were three sites, a likelihood ratio test was used to jointly assess the two resulting interaction terms. This was non-significant (p = 0.76), confirming the homogeneity and justifying a pooling of the analysis across the three centres. At baseline, OMS-HC scale scores did not differ significantly between early and late intervention groups (mean scores 46.5 versus 47.8, t = −0.95, p<0.34). Table 3 shows participants’ OMS-HC scores stratified according to intervention group. The T1 to T2 change was statistically significantly in the early group (mean change 4.3, t=4.4, p <0.0001), but not in the late group (mean change 1.5, t=1.7, p = 0.098), see Table 4. The T2 to T3 change was not significant in the early group (mean change 0.77, t=0.94, p = 0.35) but was significant in the late group (mean change 4.3, t=6.0, p < 0.0001). The difference in T1 to T2 change scores in the early versus the late group was significant, such that the null hypothesis associated with the primary analysis was rejected (mean change 4.3 versus 1.5, t=2.1, p=0.04). The same result was obtained when linear regression was used to assess the group effect with inclusion of centre as a stratification term (z = 0.197, p = 0.049). By the final assessment (T3), at which point both groups had received the intervention, scores were lower than baseline in each group and were again comparable between groups. In the early intervention group the difference between T1 and T3 was significant (mean change 3.6, t=3.6, p<0.001), as was the case in the late group (mean change 5.5, t=6.1, p<0.0001). A *t*-test comparing the final scores in the early (mean score 42.6) versus late (mean score 43.1) groups was not significant, t = −0.25, p=0.80.

**Table 3:** **OM Survey Total Score, by group (*n = *131)**

	**Sample with complete data**	**Early**		**Late**	
		***n***	**Score (95% CI)**	***n***	**Score (95% CI)**
**T1**	131	58	46.7 (44.5-48.4)	73	47.8 (45.8-49.9)
**T2**	87	36	43.6 (40.9-46.4)	51	46.8 (44.8-48.9)
**T3**	74	30	42.6 (39.8-45.4)	44	43.1 (40.6-45.5)

*The difference between T1 and T2 was statistically significant for the Early group as was the difference between T2 and T3 for the Late group. T1 to T3 differences were significant in both groups, see Table 4.

**Table 4:** Changes in unadjusted OMS-HC scores according to group, sex, and school, by study interval

	**Mean Change (SD)**	**Mean Change (SD)**	**Mean Change (SD)**
	**T1-T2**	**T2 -T3**	**T1-T3**
	**n = 87**	**n=74**	**n=74**
All participants			
Early	- 4.3 (6.0)*	- 0.8 (4.5)	- 4.4 (6.0)*
Late	- 1.5 (6.4)	- 4.3 (4.7)*	- 5.3 (6.7)*
Female			
Early	- 4.4 (5.7)*	- 1.0 (4.6)	- 4.7 (5.3)*
Late	- 2.2 (6.0)*	- 4.3 (4.9)*	- 5.7 (6.7)*
Male			
Early	- 4.1 (7.3)	- 0.1 (4.3)	- 3.4 (8.4)
Late	+ 0.6 (7.2)	- 4.4 (4.3)*	- 4.0 (6.6)
Memorial			
Early	- 3.3 (7.7)	- 1.5 (6.4)	- 9.0 (9.9) *
Late	- 0.7 (6.2)	- 4.4 (5.0)*	- 5.8 (9.8) *
Saskatchewan			
Early	- 4.8 (3.5)*	+ 0.2 (2.1)	- 3.7 (2.2)*
Late	- 3.9 (7.1)	- 2.6 (3.9)	- 6.3 (8.8)
Dalhousie			
Early	- 4.3 (6.5)*	−1.0 (4.9)	- 4.2 (6.5)*
Late	- 1.0 (6.2)	- 4.8 (4.9)*	- 4.9 (5.0)*

*Statistically significant, based on one-sample t-tests (where H_o_: change *=* 0 and α *=* 0.05).

Table 4 shows change in OMS-HC scores stratified by group, gender, and school over the course of the study. Upon receiving the contact based intervention (T1 to T2 for the early group and T2 to T3 for the late group), there was a similar reduction in OMS-HC scores in men and women and in the different settings.

The mixed model regression analysis was initially restricted to people with complete follow-up at all three time points (n=74) and included time interval (T1 to T2 versus T2 to T3), early versus late group, and indicator variables for the different universities. A likelihood ratio test again identified no group by centre interactions (p=0.85), justifying pooling across all three sites. The effect of contact-based education was assessed as a group by time interaction, which was highly significant, p<0.0001. The effect remained significant when covariates were added to the model (age, sex, and close relationship with someone with a mental illness) and with inclusion of respondents with missing data, since a mixed model can accommodate missing data under the missing at random assumption.

A separate analysis using only the Dalhousie data showed group differences from baseline to post-intervention (T1 to T2) mean difference 3.3, t=1.93, p=0.06) and baseline to one-month post-course (T1 to T3) (mean change 4.5, t=5.3, p<0.0001), consistent with the results of the multi-centre analysis.

### Effect size

Based on the unadjusted data provided in Tables 3 and 4, the contact-based education effect sizes were estimated based on change scores. The effect size associated with the first contact based education session was 0.45. Translated, this effect size indicates that 68% of pharmacy students that participated in the education session had a greater improvement in their OMS-HC scores than the average student who did not participate in the session. For the second contact based education session the impact was greater. The effect size was 0.77, indicating that 78% of those who received the education intervention had a greater change in their OMS-HC scores compared to the average score change in the other group.

## Discussion

Our study showed that mental health courses for pharmacy students using contact-based sessions reduced mental health-related stigma among pharmacy students at three pharmacy faculties in Canada. We believe this to be the first randomized study to show that such sessions diminish stigma among pharmacy students.

A minor and non-significant change in OMS-HC scores during the regular mental health education sessions and a substantial change following contact-based education sessions suggest that this intervention has a more substantial effect on reducing stigma than did regular undergraduate teaching methods. At Dalhousie University, which has a problem-based learning curriculum, the first issue brought up in the first case of the mental health module directly highlighted pharmacist-based stigmatization of an individual with schizophrenia. Each small learning group discussed this issue and was facilitated by in-room tutors. A complementary lecture discussed stigma and guided students to videos that are intended to promote their understanding of stigma from the patient’s perspective. What these cases, lectures, and videos lack is an interactive, social component that directly involves a person affected by mental illness. The results of this study point towards the added effectiveness of this kind of contact.

A previous meta-analysis examined three mediators of the effect of social contact on prejudice (a concept related to stigmatization): knowledge, anxiety reduction and empathy [23]. All three appeared to act as mediators, but knowledge had the weakest effect. Curricula that supplement their knowledge-based components with social contact may make more pronounced gains through mechanisms such as diminished anxiety and increased empathy. Allport’s optimal conditions for the effectiveness of contact in reducing prejudice, as summarized by Pettigrew and Topp [24] include: equal status between the groups; common goals; intergroup cooperation; and the support of authorities, law, or custom. Within this framework, it is not surprising that traditional rotations do not seem to robustly reduce aspects of stigma such as social distance [15]. Contact-based sessions involve social interaction characterized by greater equality than sometimes characterizes relationships between professional-providers and service-users. One of the reasons for why contact-based sessions may be more effective is that they established more equal status. Studies that have used consumer-educators (e.g. [16]) also establish greater equality and may reduce social distance as a result.

Our study had several limitations. Response rates to the baseline survey were lower at the University of Saskatchewan (40.7%) compared to the other two schools (Memorial University, 75.6%; Dalhousie University, 77.4%). The highest number of dropouts came from Memorial University and the University of Saskatchewan. Attrition can be a source of bias in this type of study. The rate of successful follow-up for the individual schools was 32.3% (Memorial), 40.0% (Saskatchewan), and 76.9% (Dalhousie). A sensitivity analysis that examined the primary outcome using only students from Dalhousie, however, found similar results. The low overall response rate may, however, diminish the generalizability of the results. For ethical reasons, we were not able to compare students choosing, or not, to participate in the study. The OMS-HC is a new tool specifically designed to measure the attitudes of health care providers towards the mentally ill. Therefore it is not yet possible to compare the effects seen in this study with those of other interventions in other populations. Another limitation is that whereas the T2 assessment occurred within a few days of the contact-based education session for the early group, the final assessment occurred one month after the late group’s session. A greater effect might have been expected in the early group because of this. However, this issue appears not to be a major one since the improvement between T2 and T3 in the late group was similar to that between T1 and T2 in the early group. As the students that had their contact-based education session would have interacted with those that had not yet participated there is the possibility that communication might have cross-contaminated the groups. If such contact had improved the attitudes of the late group at T2 it might have diluted the apparent effect of the intervention. Such cross-contamination could have contributed to a negative result, but does not diminish the credibility of the positive result that was observed in this study.

Another limitation relates to the use of an attitude scale to assess outcome. Ideally, interventions would be assessed at the level of behaviour rather than attitudes. Although OMS-HC ratings have been shown to be distinct from social desirability ratings [20], it is always possible that attitude scales are influenced by social desirability bias.

Contact-based education appears to be effective at reducing mental health related stigma in pharmacy students and findings from this study support the inclusion of this type of education in the mental health modules of pharmacy schools. Qualitative [25], uncontrolled [26] and controlled but non-randomized [16,27] interventions involving people with mental illnesses participating in pharmacy education have reported generally positive results on social distance and items assessing stigmatizing attitudes. The current study adds to this literature through its use of randomization, providing more robust evidence that the contact-based component is an active ingredient of stigma reduction.

This study was short-term. Future research should seek to determine if the effects are long lasting and, if necessary, to identify ways of maintaining or boosting the benefits over the long term. However, one prior study found that effects from a consumer-led intervention were sustained for one year [26], suggesting that such effects are not transient effects. While this study focussed on students, the population of practicing pharmacists may also be able to benefit from this type of intervention. However, the generalizability of these results to that population, or to other health professions, cannot be assumed. Furthermore, generalizability to other groups of pharmacy students and other pharmacy schools needs to be confirmed by replication of these results.

## Conclusions

Contact-based education is an effective anti-stigma intervention when incorporated into pharmacy curricula.

## Competing interests

The authors declare that they have no competing interests.

## Authors’ contributions

SBP contributed to the planning and design of the study and was involved in the data analysis. AR, LP and DMG contributed to the design and implementation of the contact-based interventions at their respective institutions and were involved from the beginning in the planning of this study. They all reviewed several drafts of the manuscript and provided comments. DMG developed the effect size illustrator employed in the analysis. AK and GM were also involved in all stages of the project. AK was the lead investigator in development of the OMS-HC scale and wrote the first draft of the research proposal for this study. GM wrote the first draft of the manuscript and carried out the statistical analysis. A revision of this initial draft, after receipt of feedback from the other authors, was written by AS. All authors read and approved the final manuscript.

## Pre-publication history

The pre-publication history for this paper can be accessed here:

http://www.biomedcentral.com/1472-6920/12/120/prepub

## Supplementary Material

Additional file 1 Opening Minds Scale for Health Care Providers (OMS-HC). Click here for file
