# Supplementary material for: Effectiveness of contact-based education for reducing mental illness-related stigma in pharmacy students
Source: BMC Med Educ. 2012 Dec 5;12:120. doi: 10.1186/1472-6920-12-120 (PMC3533989; doi:10.1186/1472-6920-12-120)
Supplement: Additional file 1 — Opening Minds Scale for Health Care Providers (OMS-HC). [file 1472-6920-12-120-S1.pdf]

Mental Health Commission of Canada

Commission de la santé mentale du Canada

Opening Minds Scale for Health Care Providers (OMS-HC)

		Strongly Disagree	Disagree	Neither Agree nor Disagree	Agree	Strongly Agree
1.	I am more comfortable helping a person who has a physical illness than I am helping a person who has a mental illness.	☐	☐	☐	☐	☐
2.	If a person with a mental illness complains of physical symptoms (e.g., nausea, back pain or headache), I would likely attribute this to their mental illness.	☐	☐	☐	☐	☐
3.	If a colleague with whom I work told me they had a managed mental illness, I would be just as willing to work with him/her.	☐	☐	☐	☐	☐
4.	If I were under treatment for a mental illness I would not disclose this to any of my colleagues.	☐	☐	☐	☐	☐
5.	I would be more inclined to seek help for a mental illness if my treating healthcare provider was not associated with my workplace.	☐	☐	☐	☐	☐
6.	I would see myself as weak if I had a mental illness and could not fix it myself.	☐	☐	☐	☐	☐
7.	I would be reluctant to seek help if I had a mental illness.	☐	☐	☐	☐	☐
8.	Employers should hire a person with a managed mental illness if he/she is the best person for the job.	☐	☐	☐	☐	☐
9.	I would still go to a physician if I knew that the physician had been treated for a mental illness.	☐	☐	☐	☐	☐
10.	If I had a mental illness, I would tell my friends.	☐	☐	☐	☐	☐
11.	It is the responsibility of health care providers to inspire hope in people with mental illness.	☐	☐	☐	☐	☐
12.	Despite my professional beliefs, I have negative reactions towards people who have mental illness.	☐	☐	☐	☐	☐
13.	There is little I can do to help people with mental illness.	☐	☐	☐	☐	☐
14.	More than half of people with mental illness don't try hard enough to get better.	☐	☐	☐	☐	☐
15.	People with mental illness seldom pose a risk to the public.	☐	☐	☐	☐	☐
16.	The best treatment for mental illness is medication.	☐	☐	☐	☐	☐
17.	I would not want a person with a mental illness, even if it were appropriately managed, to work with children.	☐	☐	☐	☐	☐
18.	Healthcare providers do not need to be advocates for people with mental illness.	☐	☐	☐	☐	☐
19.	I would not mind if a person with a mental illness lived next door to me.	☐	☐	☐	☐	☐
20.	I struggle to feel compassion for a person with mental illness.	☐	☐	☐	☐	☐

10301 Southport Lane SW, Suite 800 / 10301, Southport Lane SO, bureau 800 / Calgary, AB / T2W 1S7 / T 403.255.5808
www.mentalhealthcommission.ca / www.commissionsantementale.ca

Offices in Ottawa / Bureaux à Ottawa
